# Case report: gastric ischemia, a fatal disease of gastric pneumatosis

**DOI:** 10.1186/s12876-021-01942-y

**Published:** 2021-10-09

**Authors:** Hsueh-Chien Chiang, Chiao-Hsiung Chuang

**Affiliations:** Department of Internal Medicine, National Cheng Kung University Hospital, College of Medicine, National Cheng Kung University, No. 138, Sheng Li Road, Tainan, 704 Taiwan

**Keywords:** Gastric pneumatosis, Gastric ischemia, Endoscopy

## Abstract

**Background:**

Gastric pneumatosis indicates the presence of air within the stomach wall. The etiologies included gastric ischemia, gastric intramural infection, gastric mucosal disruption, and secondary to pneumomediastinum. Gastric ischemia is rare because of the rich collateral blood supply to the stomach.

**Case presentation:**

An 82-year-old man presented to the emergency department with a 2-day history of epigastric fullness, following by fever and low blood pressure. Chest X-ray and abdominal computed tomography revealed gastric pneumatosis at the gastric fundus. The esophagogastroduodenoscopy confirmed the ischemic change of mucosa at the gastric fundus. After antibiotics and medical management, the patient became better and was eventually discharged.

**Conclusion:**

For the diagnosis of gastric ischemia, physicians should be alert to the hints of gastric pneumatosis from X-ray and computed tomography. It is important to distinguish between gastric ischemia and the other causes of gastric pneumatosis to judge clinical management.

## Background

Gastric pneumatosis indicates the presence of air within the stomach wall. The clinical presentation ranges from benign disease to shock and mortality. Etiologies of gastric pneumatosis include gastric ischemia, gastric intramural infection, gastric mucosal disruption, and secondary to pneumomediastinum. Gastric ischemia may be local or systemic hypoperfusion, which can lead to a severe clinical emergency and even death. Gastric mucosal disruption, indicating facilitating air penetration, maybe spontaneous or iatrogenic, which is usually benign in clinical presentation. Identification of these two different etiologies is important because the clinical prognosis and management were quite different.

Gastric ischemia is uncommon because of the rich collateral blood supply to the stomach. Vascular insufficiency caused by systemic hypotension, vasculitis, or disseminated thrombo-embolism can lead to gastric ischemia. Other causes include mechanical causes such as volvulus and acute gastric distention. The diagnostic tool of gastric ischemia includes endoscopy and imaging studies. The endoscopy can estimate the severity and extent of gastric ischemia. The endoscopic finding of gastric ischemia includes diffuse or patchy gastric mucosal discoloration, loss of mucosal vascular pattern, diffuse erosions, and ulcerations. Treatment of gastric ischemia is supportive measures, including high-dose proton pump inhibitors, intravenous fluids, and antibiotics. Surgical intervention is indicated if gastric perforation is identified.

We present a case of an 82-year-old man who presented to the emergency department with a 2-day history of epigastric fullness. Chest X-ray and the computed tomography revealed gastric pneumatosis at the gastric fundus. The esophagogastroduodenoscopy confirmed the ischemic change of mucosa at the gastric fundus, and the patient was eventually discharged on conservative medical management.

## Case presentation

**Fig. 1:**
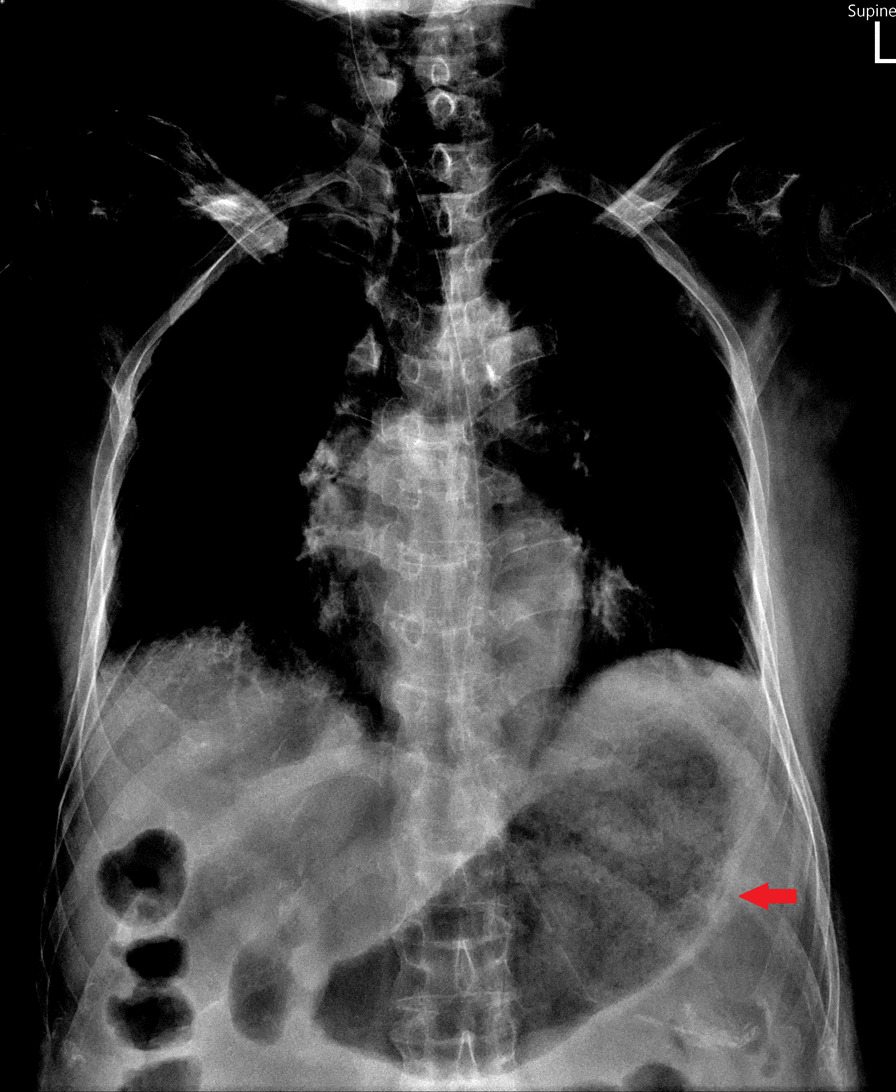
The chest X-ray showed distended stomach with thickened and blurred margin of the gastric fundus

**Fig. 2:**
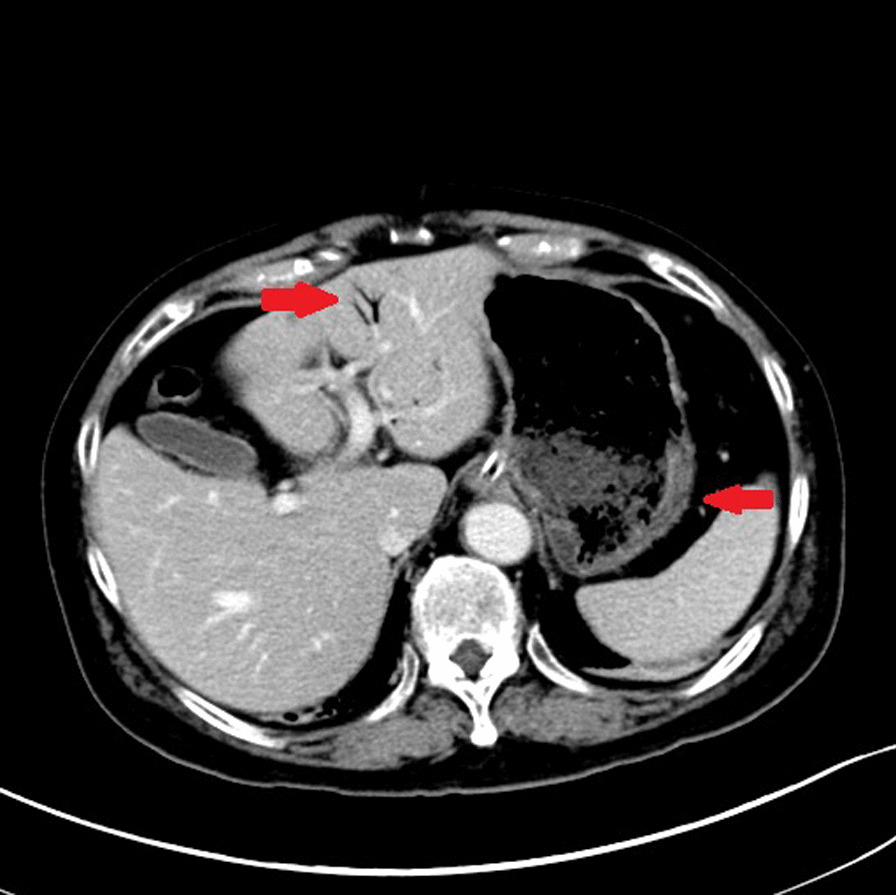
The computed tomography of the abdomen demonstrated venous air in portal veins, thickened gastric fundus wall with gastric pneumatosis

**Fig. 3:**
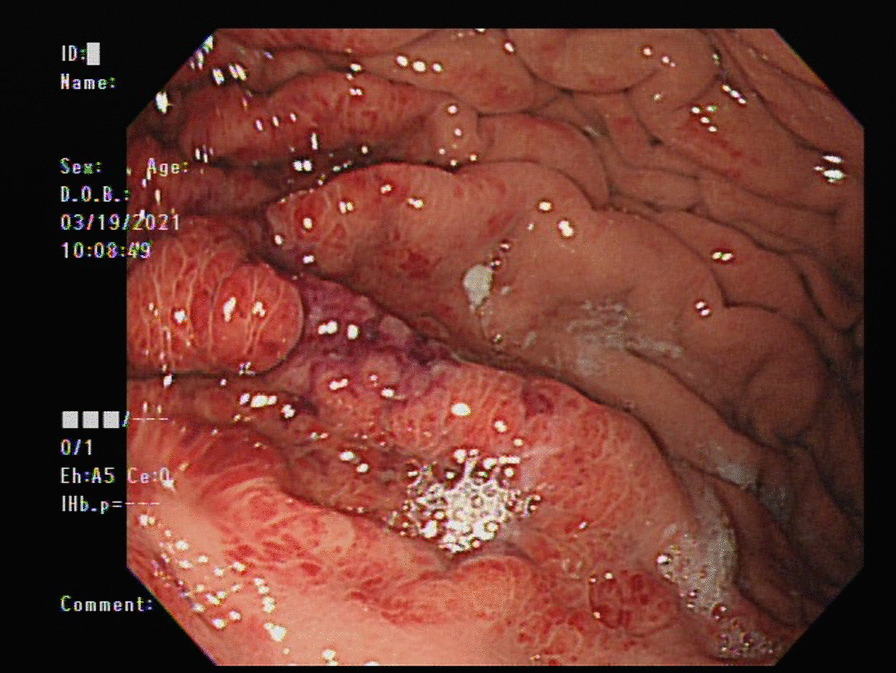
The esophagogastroduodenoscopy demonstrated focal edematous and erythematous mucosa at the fundus with diffuse shallow ulceration and hemorrhage

**Fig. 4:**
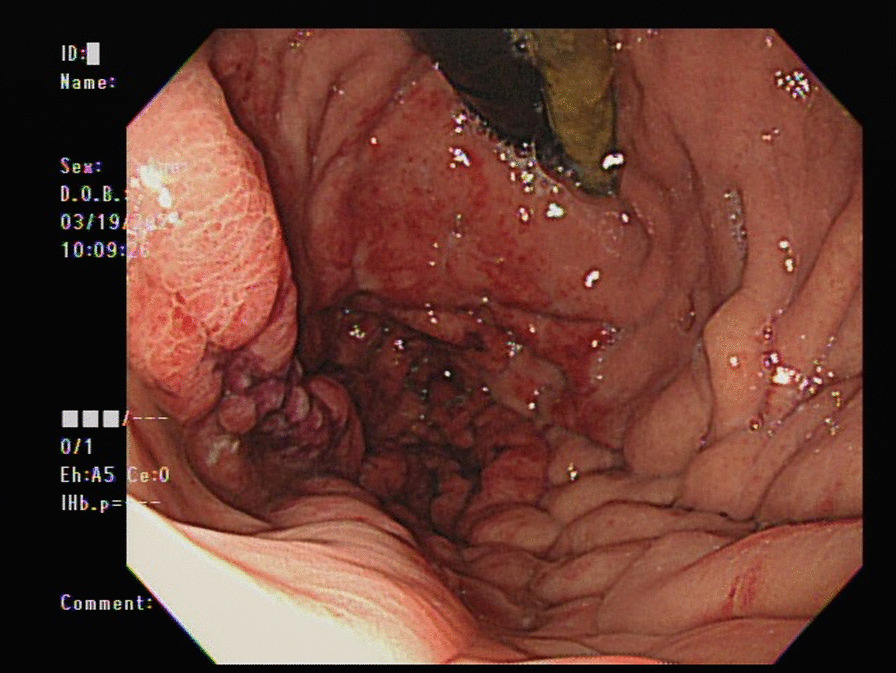
The esophagogastroduodenoscopy demonstrated a clear-cut margin of normal mucosa and ischemic mucosa at the border between the gastric fundus and the gastric body

An 82-year-old man with parkinsonism and bedridden status presented to the emergent department with a 2-day history of epigastric fullness. His physical examination revealed fever (38.5 °C), tachypnea, hypotension (86/44 mmHg), distended abdomen, and epigastric tenderness without muscle guarding or peritoneal sign. Laboratory studies demonstrated elevated white blood cell count (16,200/μL), elevated serum lactate (3.7 mmol/L), and elevated serum creatinine (1.48 mg/dL), indicating septic shock with acute kidney injury. Chest X-ray (Fig. 1) showed a distended stomach with thickened and blurred margin of the gastric fundus. Computed tomography of the abdomen (Fig. 2) showed venous air in portal veins, thickened gastric fundus wall with gastric pneumatosis. We inserted a nasogastric tube for gastric decompression. Surgical intervention was not performed in concern of old age without evidence of gastric perforation. The esophagogastroduodenoscopy demonstrated focal edematous and erythematous mucosa at the fundus with diffuse shallow ulceration and hemorrhage (Fig. 3). A clear-cut margin was noted at the border between the gastric fundus and the gastric body (Fig. 4), which was compatible with the computed tomography.

**Fig. 5:**
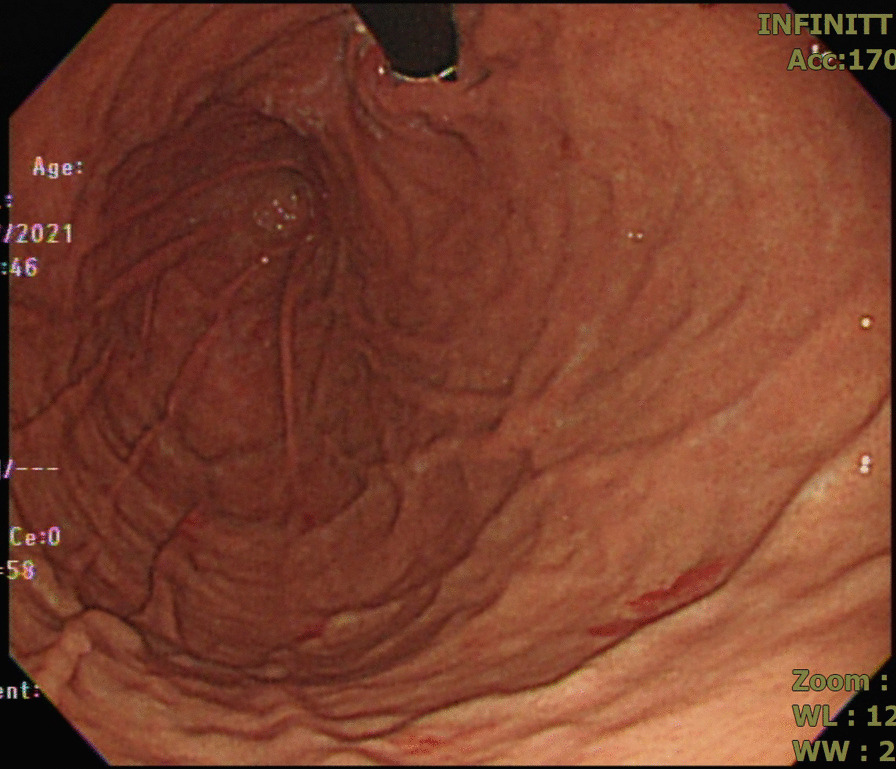
The follow-up esophagogastroduodenoscopy revealed improved gastric mucosa

We kept supportive care with intravenous fluid resuscitation, intravenous broad-spectrum antibiotics with ceftriaxone 2 g per day, and intravenous proton pump inhibitor with esomeprazole 80 mg per day. His body temperature and blood pressure became normal, and his abdominal pain gradually improved after 2 days of treatment. Follow-up serum lactate level decreased to 1.7 mmol/L. He was discharged 10 days later with an uneventful course. Five months later, a follow-up esophagogastroduodenoscopy revealed improved gastric mucosa (Fig. 5).

## Discussion and conclusion

In this report, we have presented a case of gastric ischemia with sepsis-related systemic hypotension. Because he had an initial presentation of abdominal pain and no other infection sources, gastric ischemia is thought to be the cause of sepsis. Advanced age was one of his risk factors for gastric ischemia. We sensed hints of gastric pneumatosis by chest X-ray and confirmed it via computed tomography. Because of no gastric perforation or indication for emergent surgical intervention, we performed an esophagogastroduodenoscopy and diffuse gastric ischemia was discovered.

Gastric ischemia is rare and infrequently reported in the medical literature due to the rich vascular supply to the stomach. According to previous case reports and case series, gastric ischemia occurs due to a decrease in gastric blood flow by vascular insufficiency [1]. The typical clinical presentations include nausea, vomiting, abdominal pain, abdominal distention, and upper gastrointestinal bleeding [2–4]. Image study may demonstrate portal venous air and gastric pneumatosis. Risk factors to decrease gastric and splanchnic blood flow include advancing age, smoking, atherosclerosis, diabetes, and hypertension [2]. Furthermore, systemic hypoperfusion, mechanical obstruction, or local vascular defects could be the predisposing factors of gastric ischemia [2].

Causes of gastric pneumatosis include gastric ischemia, gastric intramural infection, gastric mucosal disruption, and secondary to pneumomediastinum [3]. Gastric ischemia is risky, while other etiologies of gastric pneumatosis are less fatal. Esophagogastroduodenoscopy can help in differentiating gastric ischemia from gastric mucosal disruption. Endoscopic findings of gastric ischemia include diffuse or patchy gastric mucosal discoloration, loss of mucosal vascular pattern, diffuse erosions, and ulcerations [2, 3]. Pathology findings of gastric ischemia include capillary dilatation, mucosal edema, vascular congestion, and necrosis [3]. Gastric ischemia has a poor clinical prognosis and supportive therapy is necessary in all cases. Gastric distention can exacerbate gastric ischemia, so nasogastric tube decompression is also suggested. Surgical interventions are aimed at restoring gastric blood flow, such as incarcerated paraesophageal hernia or gastric volvulus. Gastrectomy is not necessary unless refractory bleeding, full-thickness gastric necrosis, or gastric perforation occurs [2].

In conclusion, gastric ischemia is a rare disease associated with high mortality. Gastric pneumatosis could be identified in the imaging study. Esophagogastroduodenoscopy is usually performed in diagnosis to differentiate gastric ischemia from other causes of gastric pneumatosis. Conservative treatment with fluid resuscitation, intravenous antibiotics, nasogastric tube decompression, and gastric acid suppression is suggested for all patients with gastric ischemia [2]. Some patients eventually require surgery if refractory bleeding, full-thickness gastric necrosis, or gastric perforation occurs. Evaluation for potentially treatable etiologies such as systemic hypotension, vasculitis, and thromboembolism should be sought.

## Data Availability

The datasets used and/or analyzed during the current study are available from the corresponding author on reasonable request.
